# Well-Being and Perceived Competence in School Children from 1 to 9 Class

**DOI:** 10.3390/ijerph20032116

**Published:** 2023-01-24

**Authors:** Hermundur Sigmundsson, Jan E. Ingebrigtsen, Benjamin H. Dybendal

**Affiliations:** 1 Department of Psychology, Norwegian University of Science and Technology, 7491 Trondheim, Norway; 2 Research Center for Education and Mindset, University of Iceland, 102 Reykjavík, Iceland; 3 Department of Sociology and Political Science, Norwegian University of Science and Technology, 7491 Trondheim, Norway

**Keywords:** well-being, perceived competence, reading, math, science, physical activity, gender, age, classes

## Abstract

Motivational aspects in reading, math, science and physical education are often studied on whole samples and not grouped into different classes. In our study we wish to investigate cross-sectional data across classes. Our research question wishes to investigate what class differences are there in school subject-based well-being and perceived competence? A total of 378 Icelandic pupils in classes 1–9 did answer a survey with nine items, focusing on how are you feeling at school, how do you like and how are you doing in reading, math, science and physical education. There were 163 girls (43%), and 202 boys (53%) ranging from 6 to 15 years of age (*M* = 10.86, *SD* = 2.57). The findings, for the whole sample, indicate that girls tend to like reading more than boys do. Additionally, girls feel that they are better in science compared to boys, while boys like physical education more than girls. In terms of classes, multiple items, including reading, math and science, indicated class differences, where higher classes (i.e., eighth and ninth class) tend to have lower average scores in how much they liked a certain topic, and how competent they felt. It is also of great interest that the correlation between ‘how do you like’ and ‘how are you doing’ are 0.53, 0.71, 0.66 and 0.66 for reading, math, science and physical activity, respectively. Well-being and perceived competence in all subjects correlate with each other, and well-being at school. This shows the importance of seeing the school as a holistic system, where experiences related to individual subjects coincide with the overall experience (and vice versa). The results are discussed in relation to self-perception, motivation and practice.

## 1. Introduction

Programme for International Student Assessment (PISA) measures 15-year-old students’ ability in reading, maths and science and their real-life skills [1]. The 2018 PISA report indicates that Iceland has below-average performance in both reading and science but just over the average in math [2]. The findings also indicate that Icelandic boys are doing poorly in reading (454 vs. 494), math (490 vs. 500) and science (471 vs. 479) compared to girls (OECD, 2019). In reading the OECD average difference between boys and girls is 30 points. However, Icelandic girls scored 40 points higher than the boys in reading [2]. In addition, the decreasing trend has been stable for the seven PISA assessments completed in Iceland [2]. In math, the OECD average show that boys scores 5 points higher than girls. In Iceland, girls scored 10 points higher than the boys. Lastly, the OECD results show that girls scored 8 points higher than boys in science among Icelandic students [2]. The OECD average is that girls score 2 points higher than the boys. The general findings in PISA [2] are that Iceland has a decreasing trend in reading, science and math. However, the trend indicates a more severe decrease for the boys compared to the girls. 

Based on the PISA results it is fruitful to investigate the general well-being among the Icelandic school children to understand how they are doing in school and how it might influence their motivation to perform in reading, math, science and physical education [3]. Diener et al. [4] (p. 63) defines subjective well-being as ‘a person’s cognitive and affective evaluations of his or her life’. School performance, reading, math and science are often investigated through measurements of cognitive abilities e.g [5,6,7,8]. However, studies have found that motivational factors such as perceived competence and intrinsic value have incremental value above general cognitive abilities domains such as science [9]. ‘Perceived competence (PC) refers to one’s beliefs about his or her ability to learn and executive specific skills’ [10] (p. 1). In Harter’s competence motivation theory perceived competence is a key factor [11,12]. Dicke et al. [13] (p. 2) argue that ‘students perceived competence, i.e., feeling capable with regards to the task at hand, is one of the crucial components of student motivation and is associated with positive academic outcomes, such as achievement’ [14]. This may be linked to motivational factors such as passion, grit and mindset [15,16,17,18,19,20,21]. Findings from Bailey and Phillips [22] show that intrinsic motivation predicts well-being and academic performance. Namely, both motivational aspects and well-being are important predictors of academic performance in reading, science, math and physical education.

### 1.1. Reading

Reading performance and development are often explained through cognitive measures such as decoding, language comprehension and letter-sound knowledge e.g. [5,20,23,24,25,26,27]. However, the individual must be motivated to read for reading skill to develop [28,29,30]. Reading motivation have shown to be positively related to the amount of time spent reading [31] and the amount of time spent reading has been shown to improve reading comprehension [24,25,26,27,28,29,30,31,32,33,34]. Furthermore, findings on reading performance have shown that students’ enjoyment of reading explain 18% of variance in reading performance [35]. Additionally, Cheema [35] finds that the predictive variance varies across different countries, highly academic countries have a positive relationship between gender, socioeconomic status and enjoyment and not in low-performing academic countries.

Other studies show that perceived control in reading has a negative relationship to anxiety which negatively predicts reading comprehension [36]. Namely, individuals that feel competent while reading experience less anxiety. Furthermore, self-efficacy is associated with reading more difficult texts and reading out of enjoyment, and as a result individuals with more self-efficacy perform better on reading tests compared to those who were less motivated [37]. Lastly, findings show there is a reciprocal relationship between achievement and reading motivation [38]. Namely, both reading achievement and reading performance predicted each other at two timepoints among twins, indicating a genetic factor in this reciprocal relationship [38]. Consequently, motivational aspects are associated with reading performance [39]. Kuzyk et al. [40] (p. 3) argues that girls perceive themselves more positively in reading and writing than in subjects such as math and science. In relation to perceived competence in reading, girls tend to rate themselves higher than boys [41] (p. 110).

### 1.2. Math

General cognitive abilities, spatial abilities and processing speed are often used to predict academic achievements in mathematics [6]. However, findings on motivational variables such as academic self-concept show that having high perceived math competence predicts academic performance in math, and enjoyment predicts higher perceived math competence [42]. Additionally, Christensen and Knezek [43] finds that enjoyment and confidence have a reciprocal relationship, but that enjoyment and confidence decrease over the years. Namely, individuals that perceive math as fun engages more in math activities [44]. Engagement in math specific activities have shown to increase math confidence [43]. Hence, when confidence increases, the individuals have shown to experience more enjoyment in that domain. As a result, the reciprocal relationship between enjoyment and perceived achievement have shown to increase performance in math [45]. Researchers have argued that boys perceive themselves more positively in math than girls [46,47]. This might be related to gender differences in achievement-related beliefs [48], which may be linked to passion for achievement. Research has indicated gender differences in passion for an area/theme/skill; males have a higher score [15,16]. Golding et al. [49] found, in their study, that girls reported enjoying mathematic less than boys.

### 1.3. Science

Like reading and math, engagement in science requires individual motivation and interest [7]. Areepattamannil and colleagues [50] show that important predictors of science achievement include motivational beliefs, self-efficacy and enjoyment. Both enjoyment and the experienced competence have been linked to increased psychological well-being (i.e., need satisfaction; [51]). Furthermore, studies show that among students in Hong-Kong, enjoyment and self-efficacy in science predict higher science achievements [52]. Namely, believing in one’s capabilities and enjoyment of a subject are related to higher performance. Next, science enjoyment is related to science interest, which mediate the relationship between enjoyment and embedded interest in science (i.e., desire to further engage in specific domain topics; [53]). Consequently, findings show that both perceived competence and enjoyment are reciprocal, meaning that an increase in enjoyment might improve perceived performance and the other way around [42].

### 1.4. Physical Education

Children and adolescents are advised to spend 60 min in moderate-vigorous activity each day [54]. Furthermore, physical education contributes to development of motor skills and physical fitness among children [55]. However, moderate-vigorous activity has shown to decrease in the higher grades [56]. Nonetheless, studies show that experienced competence in physical education explained both engagement in physical activity during physical education, but also outside of school [57]. Still, there was no differences in levels of physical activity based on the reported levels of enjoyment among the students [57]. Additionally, Gråstén and colleagues [58] find that task climates in physical education are an important predictor of enjoyment, which further predicts perceived competence. Namely, it is important to nurture climates in physical education that focus on learning, since it predicts higher enjoyment and perceived competence among children. Hence, enjoyment and perceived competence predict higher engagement in physical education [59]. Lastly, findings show that boys report more time spent on physical activity, perceived competence and enjoyment than girls did [57]. Ruble et al. [41] (p. 110) found, in relation to physical competence, that boys are much more likely to rate themselves higher than girls.

Motivational aspects in reading, math, science and physical education are often studied on whole samples and not grouped into different classes. In our study we wish to investigate cross-sectional data across classes. Our main research question wishes to investigate what class differences are there in school subject-based well-being and perceived competence? Our secondary aim is to analyse the relationship between the different factors. There it is of interest to see the relationship between ‘how do you like’ and ‘how are you doing’ in the subjects reading, math, science and physical education.

## 2. Method

### 2.1. Participants

At the beginning the students reported their age, gender and what grade they were in. The study consists of 378 Icelandic pupils from 1–9 grade. The children ranged from 6 to 15 years of age (*M* = 10.86, *SD* = 2.57). There were 163 girls (43%), and 202 boys (53%). The girls had a mean age of 10.92 (*SD* = 2.54), and the boys had a mean age of 10.81 (*SD* = 2.59). Among the respondents, 13 chose not to report their gender (3%). The data collection was carried out in May 2021. The children participating in the study come from Vestmanna Island which is a group of 14 small Icelandic islands off Iceland’s southern shore. Heimaey is the largest and only inhabited island in which the town Vestmannaeyjar is located. The population of the islands is around 4.000. The sample included children in a wide range of socio-economic backgrounds [20] (p. 2). The school curriculum is similar to the Nordic countries as Norway. From the autumn 2021 the Vestmanna Islands elementary school is part of the ‘Ignition approach’ (Kveikjum neistann) which has focused on increasing achievement and well-being of the students. The project builds on the theories to Ericsson ‘deliberate practice’ [30], Csikszentmihalyi ‘flow’ [60] and Sigmundsson ‘motivational factors’ [15,17]. The approach has much focus on daily physical activity, training hour with challenges in relation to skill and passion hour. In the passion hour the children have the possibility to choose subjects to work on in the end of the day. By choosing subjects it is possible to increase the passion for achievement and therefore the motivation and well-being [15,17,18,19,20].

### 2.2. Measures

#### 2.2.1. Well-Being and Perceived Competence

Respondents answered a survey, with nine items totally. Four items regarding their subjective perceived competence ‘how are you doing’ in math, science, reading and physical education (see Table 1). Furthermore, the respondents answered five questions regarding their subjective well-being ‘how are you feeling at school’ and ‘how do you like’ reading, math, science and physical education (see Table 1). The students answered the questions with a use of emojis, ranging from 1–5 Likert-scale, where 1 corresponds to a very sad face, and 5 corresponds to a very happy face (3 was a neutral face). The questionnaire was developed by the Research group for learning and skill development at NTNU. The perceived competence questions are built on Harter [11,12] theory and methodology. The well-being questions were developed by our research group.

#### 2.2.2. Test-Retest Reliability

The scales used to measure well-being and perceived competence showed a good test-retest reliability. ICCs between the test scores and retest scores show a range from 0.87 to 1.00 (See Table 2). The test-retest included seven students who answered 1 week apart. See Table 2 for means and standard deviations for the included variables. The test-retest participants had a mean age of 14.75 (*SD* = 0.50).

### 2.3. Procedure

The study was completed in accordance with the Declaration of Helsinki. The data collection was carried out as a part of the school curriculum. Passive consent from the participants was confirmed to be sufficient from the Data Protection Authority, because no sensitive personal data were collected. The information registered about the participants was anonymous (only age, gender and class). The children and adolescents were assessed in a group setting at the school campus (during normal school hours). The teachers were the ones responsible for administrating the survey.

### 2.4. Data Analysis

The data were analysed with the use of SPSS version 28 for windows (SPSS Inc., Chicago, IL, USA). Scores were entered manually by one of the authors and prepared for analysis. First, we did an independent sample *t*-test to test gender differences across the nine variables. Next, to check how boys and girls like school across classes, a split by gender one-way ANOVA was used to measure class differences. A one-way MANOVA was performed to investigate mean differences in the variables across the nine classes. To correct for multiple comparisons in the MANOVA, Bonferroni post-hoc was used. Statistical significance was set to *p* < 0.05. Since this study is exploratory, we used Pearson correlation to investigate the relationship between the variables included in the survey. Missing values was replaced with the mean from the respondents group belonging (grade or gender if class was missing).

## 3. Results

### 3.1. Descriptive Statistics

An independent sample *t*-test of the whole sample showed that there were mean differences in test scores based on gender in three of the nine items (see Table 3). Girls (*M* = 3.77, *SD* = 1.06) reported that they liked reading more than boys (*M* = 3.38, *SD* = 1.27) did, *t*(363) = 3.10, *p* < 0.01. Additionally, girls (*M* = 4.06, *SD* = 1.03) felt that they did well in science more than boys (*M* = 3.77, *SD* = 1.10) did, *t*(363) = 2.56, *p* < 0.05). Boys (*M* = 4.42, *SD* = 1.00) reported that they liked sports and swimming on average more than girls (*M* = 4.15, *SD* = 1.15) did, *t*(362) = −2.42, *p* < 0.05. There were no significant mean differences in how they felt about school and how they liked maths and science. Lastly, there were no significant mean differences in how they felt they were doing in reading, maths, and sports/swimming.

### 3.2. Mean Differences across Classes on ‘How Are You Feeling at School’ (Split by Gender)

**Figure 1:**
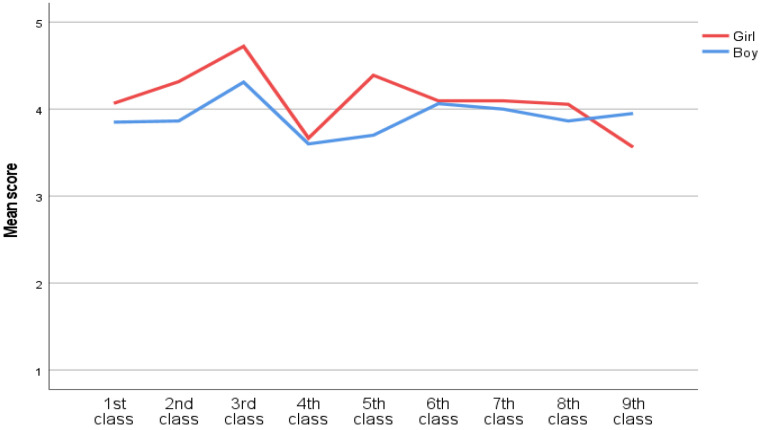
How are you feeling at school?

A one-way ANOVA split by gender was run to test for mean class differences among each gender in ‘how are you feeling at school’ (See Figure 1). The one-way ANOVA showed significant differences across classes among the girls, *F*(8, 155) = 2.41, *p* = 0.018. Among the boys there were no significant differences between classes in ‘how are you feeling at school’, *F*(8, 194) = 0.79, *p* = 610. Girls in third class (*M* = 4.72, *SD* = 0.58) significantly are feeling better at school than girls in ninth class (*M* = 3.56, *SD* = 1.03). There were no significant differences in the rest of the classes.

### 3.3. Class Differences in Test Scores across Classes in Group as Whole

**Figure 2:**
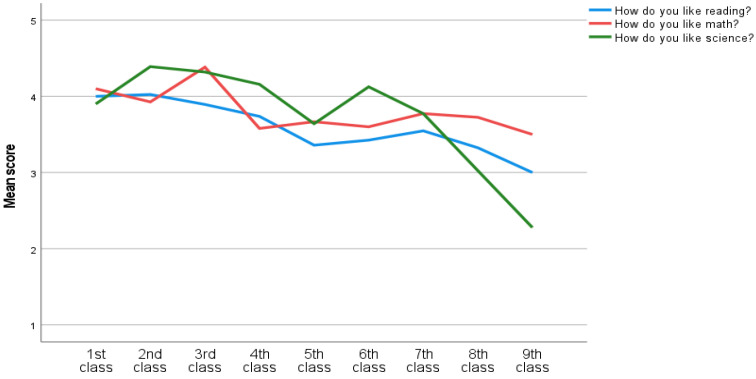
How do you like reading, math and science?

**Figure 3:**
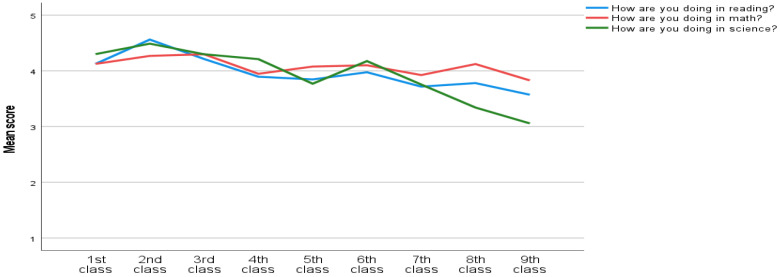
How are you doing in reading, math and science?

**Figure 4:**
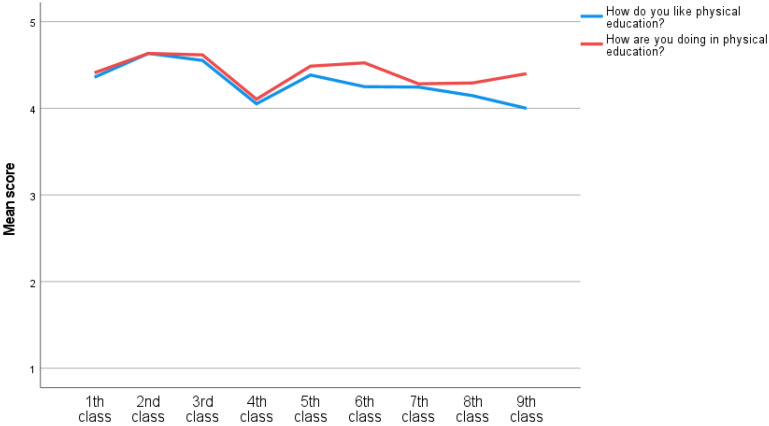
How do you like and how are you doing in physical education?

A one-way MANOVA was used to investigate the differences in the nine classes among the eight questions. The multivariate analysis indicated significant class differences in the eight test scores, *F*(72, 306) = 2.65, *p* < 0.001, Willk’s Λ = 0.59, partial η^2^ = 0.07. An inspection of the univariate analysis showed that there were significant differences in liking and perceived competence in reading and science among the nine classes. There was also significant difference in liking math among the nine classes (See Figure 2, Figure 3 and Figure 4). Since multiple comparisons were used, Bonferroni post-hoc was used to investigate mean differences in how they like reading, math and science. Lastly, post-hoc was used to investigate mean differences in how they are doing in reading and science.

There were no significant differences in ‘how do you like’ and ‘how are you doing’ in physical education and ‘how are you doing in math’ (See Figure 2, Figure 3 and Figure 4). Hence, there was found to be no need to investigate these in the post-hoc analysis.

#### 3.3.1. Reading WB (Well-Being), “How Do You Like Reading?”

An inspection of the univariate analysis showed that there were significant differences among the classes on Reading WB, *F*(8, 370) = 3,61, *p* < 0.001. The post-hoc test showed that ninth (*M* = 3.00, *SD* = 1.21) classes scored significantly lower than first (*M* = 4.00, *SD* = 1.49), second (*M* = 4.02, *SD* = 1.01) and third (*M* = 3.89, *SD* = 1.11) classes in Reading WB scores (See Figure 2). To explain, ninth class liked reading on average less than first, second and third classes did.

#### 3.3.2. Reading PC (Perceived Competence), “How Are You Doing in Reading?”

The nine classes differed significantly in Reading PC, *F*(8, 370) = 3,78, *p* < 0.001. The post-hoc analysis showed that second (*M* = 4.56, *SD* = 0.63) classes scored significantly higher than fifth (*M* = 3.85, *SD* = 1.04), seventh (*M* = 3.72, *SD* = 1.01), eighth (*M* = 3.78, *SD* = 0.79) and ninth (*M* = 3.57, *SD* = 0.87) classes in Reading PC (See Figure 3). Results show that second classes felt that they on average were doing better in reading than fifth, seventh, eighth and ninth classes did.

#### 3.3.3. Math WB, “How Do You Like Math?”

The univariate analysis indicated that there were significant differences across classes on how the students liked math, *F*(8, 370) = 2.15, *p* = 0.031. However, an inspection of the post-hoc results indicated no significant differences between the classes.

#### 3.3.4. Science WB, “How Do You Like Science?”

The nine classes differed in Science WB, *F*(8, 370) = 14.75, *p* < 0.001. In Science WB, first (*M* = 3.90, *SD* = 1.48) class had significantly higher scores then eighth (*M* = 3.03, *SD* = 1.31) and ninth (*M* = 2.28, *SD* = 1.14) classes. Second (*M* = 4.39, *SD* = 1.10), third (*M* = 4.32, *SD* = 0.96), fourth (*M* = 4.16, *SD* = 0.96), sixth (*M* = 4.13, *SD* = 0.72) and seventh (*M* = 3.77, *SD* = 0.82) classes also had significantly higher scores than eighth and ninth classes. Fifth (*M* = 3.64, *SD* = 1.20) class had significantly higher scores then ninth class (See Figure 2).

#### 3.3.5. Science PC, “How Are You Doing in Science?”

Results showed that the nine classes differed in Science PC, *F*(8, 370) = 9.48, *p* < 0.001. In Science PC, first (*M* = 4.30, *SD* = 1.32) class scored significantly higher than eighth (*M* = 3.34, *SD* = 1.04) and ninth (*M* = 3.06, *SD* = 1.12) classes. Secondly, second (*M* = 4.49, *SD* = 0.87) class had significantly higher scores than fifth (*M* = 3.77, *SD* = 0.96), seventh (*M* = 3.76, *SD* = 0.78), eighth and ninth classes. Thirdly, third (*M* = 4.30, *SD* = 0.98) class had significantly higher scores than eighth and ninth classes. Fourthly, the fourth (*M* = 4.21, *SD* = 0.92) class had significantly higher scores than ninth class. Next, the sixth (*M* = 4.17, *SD* = 0.75) class had significantly higher scores than the eighth and ninth classes. Lastly, the seventh (*M* = 3.75, *SD* = 0.78) class had significantly higher scores then the ninth class (See Figure 3).

### 3.4. Correlations between Variables

First, a Pearson’s correlational analysis (See Table 4) showed that Math PC highly correlated with Math WB, *r* = 0.71, *p* <. 01. Additionally, there was a strong significant correlation between PE WB and PE PC, *r* = 0.66, *p* < 0.01. PE PC had a weak significant correlation with Reading WB, *r* = 0.19, *p* < 0.01 and lastly, PE PC had a weak significant correlation with Science WB, *r* = 0.18, *p* < 0.01.

## 4. Discussion

The findings indicate that girls tend to like, and feel they are more competent in, theoretical subjects, reading, math and science, than boys do. Boys tend to like physical education, and feel more competent, than girls do. In terms of classes, multiple items, including reading, math and science, indicated class differences, where higher classes tend to have lower average scores in how much they liked a certain topic, and how competent they felt.

The class differences among girls were found in how they are feeling at school in general. Among the girls, the lower class tends to have higher average scores in the aforementioned subject. Boys had no class differences in how they are feeling about school. It is interesting to see the development from the 8 class to the 9 class for the genders. Boys scores increase and girls decrease.

It is also of great interest to see that the correlation between ’How do like’ and ’How are you doing’ are 0.53, 0.71, 0.66 and 0.66 for reading, math, science and physical activity, respectively. Well-being and perceived competence in all subjects correlate with each other, and well-being at school. This shows the importance of seeing the school as a holistic system, where experiences related to individual subjects coincide with the overall experience (and vice versa) [20].

### 4.1. Gender Differences in Reading, Science and Physical Education

Results indicate mean differences in how much girls liked reading compared to boys (see Table 3). This study finds that girls reported liking reading more than the boys did. Former research has indicated that girls tend to have a more positive reading attitude, read more frequently and have better reading comprehension compared to boys [61]. As a result, girls tend to be more motivated to read due to the interplay between comprehension, positive attitude, and frequent reading. Namely, such motivation may be influenced by the teacher’s perception where Boerma et al. [62] finds that girls’ concept of reading and value of reading tend to be associated with teacher perceptions. Consequently, more value of reading could motivate frequent reading, which could improve reading comprehension through consistent practice [63,64]. The results suggest that girls feel more competent in science compared to boys (see Table 3). According to the PISA results, girls also tend to score higher in science compared to boys [2]. Additionally, studies by Britner et al. [65] suggest that girls have higher grades and self-efficacy in earth sciences. However, girls seem to have lower self-confidence and as a result assert less value to maths and physical sciences than boys do [66]. Other findings have also suggested that girls’ performance in science-related fields tend to be lower compared to boys [67]. Still, the findings indicate that girls in 1–9th class tend to feel more competent than boys do, and that STEM-related stereotypes might influence girls’ career-related choices later in life [68,69]. Consistent with former findings boys tend to perceive more competence in physical education compared to girls [70,71] (see Table 3). The gender difference might influence time spent on physical activity outside of school for each gender. Namely, boys tend to feel more competent in physical education, and as a result spend more time being physically active outside of school [57]. Since the boys perceive more competence in physical education compared to girls, they might be more engaged. That is, engagement and perception of competence is reciprocal—being exposed to more physical activity might strengthen the perception of competence, and the other way around [59]. Findings by Shen and colleagues [72], indicate that parental support might influence physical education enjoyment among girls with low perception of physical competence. In fact, increasing enjoyment through parental support might benefit the perceived physical competence among girls [55]. Consequently, having lower perceived competence might influence the engagement in physical education among the girls [56].

### 4.2. Class Differences

The results from this study indicate that both perceived competence (‘how are you doing’) and well-being (’how do you like’) of science, math and reading is decreasing through classes among Icelandic school children (see Figure 2, Figure 3 and Figure 4). Similarly, the findings from PISA [2], show that Iceland has a decreasing trend in reading, math and science performance. Former findings have established the importance of perceived competence and enjoyment in academical performance (e.g; [35,36,45,50]. To explain, the relationship between perceived competence and well-being is reciprocal [73]. That is, when the individual experiences more enjoyment in a specific domain, they are more likely to engage in the domain. Thus, more engagement can increase the performance, and feeling of mastery which is strengthening perceived competence [39]. Consequently, when the individuals feel less competent, they might also enjoy the specific domain less. As a result, Icelandic students might disengage with activities related to reading, math and science. Next, the disengagement can influence amount of time spent practicing and decrease performance [29,30,63]. Consequently, a decrease in either well-being or perceived competence might demotivate the student to engage in the specific activity. This may be linked to decrease in important motivational factors as passion for achievement, grit and growth mindset [15,16,17]. The teachers have an important role in keeping the child’s passion or strong interest for an area/theme/skill through the school years. With increasing passion, grit will also increase. At the same time the parents/guardians have an important role in their children’s education [20]. It can be said that growth mindset is a belief in one’s own abilities and potential. Growth mindset has proven to be one of the keys to success [74] and is probably an underlying factor for passion and grit [16]. Growth mindset should be all encompassing in our society. Both within the walls of the family, in school and sports. Schools should also place great emphasis on increasing the children’s mastery experiences. They can do that by providing the right challenges in relation to competence [20,60].

### 4.3. Limitations

There are some limitations in this study. Firstly, the data are not longitudinal and only cross-sectional. Thus, no causal relationship between class belonging and, perceived competence and well-being can be made. Secondly, the number of observations in each class is skewed, and thus the mean group differences should be interpreted with caution. 

### 4.4. Implications and Future Studies

The findings (Figure 2, Figure 3 and Figure 4) suggest that well-being and perceived competence, in school, is decreasing among Icelandic youth. Future studies should investigate the benefits of interventions to improve motivation in the specific fields, overall well-being. We should aim for increasing mastery and the feeling of ’I can’ [20]. Furthermore, others should investigate the same research question with a bigger sample, and possibly with a longitudinal design. Lastly, others can investigate the benefits from using scales that measure well-being and perceived competence in the specific domains, with more than one item.

## 5. Conclusions

For the whole sample, girls tend to like reading more than boys do. Additionally, girls perceive that they are better in science compared to boys, while boys like physical education more than girls. In terms of classes, multiple items, including reading, math and science, indicated class differences, whereas higher classes (i.e., eighth and ninth class) tend to have lower average scores in how much they liked a certain topic, and how competent they felt. It is of great interest to see that the correlation between ’How do like’ and ’How are you doing’ in all subjects correlate with each other, and well-being at school. This shows the importance of seeing the school as a holistic system, where experiences related to individual subjects coincide with the overall experience (and vice versa).

## Figures and Tables

**Table 1:** Nine questions used in survey.

School.	How Are You Feeling at School?
Reading 1.	How do you like reading?
Reading 2.	How are you doing in reading?
Math 1.	How do you like math?
Math 2.	How are you doing in math?
Science 1.	How do you like science?
Science 2.	How are you doing in science?
Physical Education (PE) 1.	How do you like physical education?
Physical Education (PE) 2.	How are you doing in physical education?

**Table 2:** Means and standard deviations of the test and retests scores (N=7).

	Test Score		Retest Score		ICC
	M	SD	M	SD	
School	4.00	1.00	4.14	0.90	0.96
Reading 1	3.14	1.22	3.43	0.79	0.93
Reading 2	3.57	0.79	3.29	1.11	0.92
Math 1	3.43	1.13	3.57	0.98	0.97
Math 2	4.14	1.07	3.86	1.46	0.96
Science 1	2.43	0.98	2.29	0.95	0.96
Science 2	3.29	0.76	3.29	0.76	1.00
PE 1	3.86	0.69	3.86	0.90	0.87
PE 2	4.43	0.79	4.57	0.79	0.94

Note, ICCs = intraclass correlation coefficients.

**Table 3:** Mean and standard deviations group as whole.

	Group as Whole (*N* = 378)	Boys (*n* = 202)	Girls (*n* = 163)	
	Mean (SD)	Mean (SD)	Mean (SD)	*p* ^a^
School	4.01 (1.01)	3.93 (1.03)	4.12 (0.93)	0.075
Reading 1	3.55 (1.20)	3.39 (1.27)	3.77 (1.06)	0.002 **
Reading 2	3.94 (1.02)	3.89 (1.05)	4.03 (0.96)	0.180
Math 1	3.81 (1.26)	3.81 (1.28)	3.87 (1.18)	0.680
Math 2	4.07 (1.00)	4.08 (1.04)	4.10 (1.13)	0.825
Science 1	3.73 (1.25)	3.68 (1.21)	3.77 (1.31)	0.527
Science 2	3.89 (1.08)	3.77 (1.10)	4.06 (1.03)	0.011 *
PE 1	4.29 (1.08)	4.42 (1.00)	4.15 (1.15)	0.015 *
PE 2	4.42 (0.90)	4.50 (0.88)	4.36 (0.91)	0.183

Note: ^a^ Independent sample *t*-test. * *p* < 0.05, ** *p* < 0.01. 1 = ‘*How do you like*’, 2= ‘*How are you doing in*’.

**Table 4:** Bivariate correlations for group as whole.

	(1)	(2)	(3)	(4)	(5)	(6)	(7)	(8)	(9)
School (1)									
Reading 1 (2)	0.49 **								
Reading 2 (3)	0.39 **	0.53 **							
Math 1 (4)	0.48 **	0.53 **	0.42 **						
Math 2 (5)	0.40 **	0.37 **	0.54 **	0.71 **					
Science 1 (6)	0.39 **	0.50 **	0.40 **	0.40 **	0.33 **				
Science 2 (7)	0.36 **	0.43 **	0.52 **	0.35 **	0.46 **	0.66 **			
PE 1 (8)	0.31 **	0.30 **	0.33 **	0.37 **	0.36 **	0.26 **	0.27 **		
PE 2 (9)	0.30 **	0.19 **	0.33 **	0.31 **	0.35 **	0.18 **	0.28 **	0.66 **	

Note: Pearson bivariate correlations between every question in the survey (two-tailed). ** *p* < 0.01, 1 = ‘*How do you like*’, 2 = ‘*How are you doing in*’.

## Data Availability

The datasets used and analyzed in the current study are available from the corresponding author upon a reasonable request.

## References

[1] OECD. Organisation for Economic Co-Operation and Development (2016). PISA 2015 Results: Excellence and Equity in Education.

[2] OECD (2019). PISA 2018 Assessment and Analytical Framework.

[3] Ansari W.E., Stock C. (2010). Is the health and wellbeing of university students associated with their academic performance? Cross sectional findings from the United Kingdom. Int. J. Environ. Res. Public Health.

[4] Diener E., Lucas R.E., Oishi S. (2002). Subjective well-being: The science of happiness and life satisfaction. Handbook of Positive Psychology.

[5] Hulme C., Snowling M.J. (2011). Children’s reading comprehension difficulties: Nature, causes, and treatments. Curr. Dir. Psychol. Sci.

[6] Rohde T.E., Thompson L.A. (2007). Predicting academic achievement with cognitive ability. Intelligence.

[7] Singh K., Granville M., Dika S. (2002). Mathematics and science achievement: Effects of motivation, interest, and academic engagement. J. Educ. Res..

[8] Spinath B., Spinath F.M., Riemann R., Angleitner A. (2003). Implicit theories about personality and intelligence and their relationship to actual personality and intelligence. Personal. Individ. Differ.

[9] Spinath B., Spinath F.M., Harlaar N., Plomin R. (2006). Predicting school achievement from general cognitive ability, self-perceived ability, and intrinsic value. Intelligence.

[10] Morgado L.D.S., Martelaer K.D., Sääkslahti A., Howells K., Barnett L.M., D’Hondt E., Costa A.M., Jidovtseff B. (2023). Face and Content Validity of the Pictorial Scale of Perceived Water Competence in Young Children. Children.

[11] Harter S. (1978). Effectance Motivation Reconsidered: Toward a Developmental Model. Hum. Dev..

[12] Harter S. (1982). The perceived competence scale for children. Child Dev..

[13] Dicke A.L., Safavian N., Gao Y., Eccles J.S. Do I Belong? Gender, Perceived Competence and the Development of Field Belonging for Physics Undergraduates. *Grantee Submission*. https://eric.ed.gov/?id=ED619061.

[14] Wigfield A., Eccles J.S., Fredricks J.A., Simpkins S., Roeser R.W., Schiefele U., Lerner R.M., Lamb M. (2015). Development of achievement motivation and engagement. Handbook of Child Psychology and Developmental Science.

[15] Sigmundsson H., Haga M., Hermundsdottir F. (2020). Passion, grit and mindset in young adults: Exploring the relationship and gender differences. New Ideas Psychol..

[16] Sigmundsson H., Guðnason S., Jóhannsdóttir S. (2021). Passion, grit and mindset: Exploring gender differences. New Ideas Psychol..

[17] Sigmundsson H., Haga M., Elnes M., Dybendal B.H., Hermundsdottir F. (2022). Motivational Factors Are Varying across Age Groups and Gender. Int. J. Environ. Res. Public Health.

[18] Sigmundsson H., Dybendal B.H., Loftesnes J.M., Olafsson B., Grassini S. (2022). Passion a key for success: Exploring motivational factors in football players. New Ideas Psychol..

[19] Sigmundsson H., Dybendal B.H., Grassini S. (2022). Motion, relation, and passion in brain physiological and cognitive aging. Brain Sci..

[20] Sigmundsson H., Thórsdóttir H.S., Njálsdóttir H.R., Hjaltalín S.T. (2022). Reading: From the Simple to the Complex. Brain Sci..

[21] Sigmundsson H. (2021). Passion, grit and mindset in the ages 14 to 77: Exploring relationship and gender differences. New Ideas Psychol..

[22] Bailey T.H., Phillips L.J. (2016). The influence of motivation and adaptation on students’ subjective well-being, meaning in life and academic performance. High. Educ. Res. Dev..

[23] Hjetland H.N., Lervåg A., Lyster SA H., Hagtvet B.E., Hulme C., Melby-Lervåg M. (2019). Pathways to reading comprehension: A longitudinal study from 4 to 9 years of age. J. Educ. Psychol..

[24] Nation K. (2019). Children’s reading difficulties, language, and reflections on the simple view of reading. Aust. J. Learn. Difficulties.

[25] Sigmundsson H., Eriksen A.D., Ofteland G.S., Haga M. (2017). Sound knowledge: Exploring gender differences in children when they start school regarding knowledge of large letters, small letters, sound large letters, and sound small letters. Front. Psychol..

[26] Sigmundsson H., Dybfest Eriksen A., Ofteland G.S., Haga M. (2018). Gender gaps in letter-sound knowledge persist across the first school year. Front. Psychol..

[27] Sigmundsson H., Haga M., Ofteland G.S., Solstad T. (2020). Breaking the reading code: Letter knowledge when children break the reading code the first year in school. New Ideas Psychol..

[28] McGeown S.P., Duncan L.G., Griffiths Y.M., Stothard S.E. (2015). Exploring the relationship between adolescent’s reading skills, reading motivation and reading habits. Read. Writ..

[29] Sigmundsson H., Trana L., Polman R., Haga M. (2017). What is trained develops! theoretical perspective on skill learning. Sports.

[30] Ericsson A., Pool R. (2016). Peak: Secrets from the New Science of Expertise.

[31] Schiefele U., Schaffner E., Möller J., Wigfield A. (2012). Dimensions of reading motivation and their relation to reading behavior and competence. Read. Res. Q..

[32] Locher F., Pfost M. (2020). The relation between time spent reading and reading comprehension throughout the life course. J. Res. Read..

[33] Stutz F., Schaffner E., Schiefele U. (2016). Relations among reading motivation, reading amount, and reading comprehension in the early elementary grades. Learn. Individ. Differ..

[34] Pfost M., Dörfler T., Artelt C. (2013). Students’ extracurricular reading behavior and the development of vocabulary and reading comprehension. Learn. Individ. Differ..

[35] Cheema J.R. (2018). Adolescents’ enjoyment of reading as a predictor of reading achievement: New evidence from a cross-country survey. J. Res. Read..

[36] Zaccoletti S., Altoè G., Mason L. (2020). Enjoyment, anxiety and boredom, and their control-value antecedents as predictors of reading comprehension. Learn. Individ. Differ..

[37] Mucherah W., Yoder A. (2008). Motivation for reading and middle school students’ performance on standardized testing in reading. Read. Psychol..

[38] Malanchini M., Wang Z., Voronin I., Schenker V.J., Plomin R., Petrill S.A., Kovas Y. (2017). Reading self-perceived ability, enjoyment and achievement: A genetically informative study of their reciprocal links over time. Dev. Psychol..

[39] Froiland J.M., Oros E. (2014). Intrinsic motivation, perceived competence and classroom engagement as longitudinal predictors of adolescent reading achievement. Educ. Psychol..

[40] Kuzyk O., Gendron A., Lopez L.S., Bukowski W.M. (2022). Gender and contextual variations in self-perceived cognitive competence. Front. Psychol..

[41] Ruble D.N., Greulich F., Pomerantz E.M., Gochberg B. (1993). The role of gender-related processes in the development of sex differences in self-evaluation and depression. J. Affect. Disord..

[42] Pinxten M., Marsh H.W., De Fraine B., Van Den Noortgate W., Van Damme J. (2014). Enjoying mathematics or feeling competent in mathematics? Reciprocal effects on mathematics achievement and perceived math effort expenditure. Br. J. Educ. Psychol..

[43] Christensen R., Knezek G. (2020). Indicators of middle school students’ mathematics enjoyment and confidence. Sch. Sci. Math..

[44] Duda G., Garrett K. (2008). Blogging in the physics classroom: A research-based approach to shaping students’ attitudes toward physics. Am. J. Phys..

[45] Fisher P.H., Dobbs-Oates J., Doctoroff G.L., Arnold D.H. (2012). Early math interest and the development of math skills. J. Educ. Psychol..

[46] Stipek D.J. (1984). Sex differences in children’s attributions for success and failure on math and spelling tests. Sex Roles.

[47] Bandura A. (1997). Self-Efficacy: The Exercise of Control.

[48] Eccles J.S. (1987). Gender roles and women’s achievement-related decisions. Psychol. Women Q..

[49] Golding J., Hill M.J., Custodio I., Grima G. (2022). Gender, self-perception, and mathematics: The 2020 England, Wales, and Northern Ireland PISA Field Trial. Res. Math. Educ..

[50] Areepattamannil S., Freeman J.G., Klinger D.A. (2011). Influence of motivation, self-beliefs, and instructional practices on science achievement of adolescents in Canada. Soc. Psychol. Educ..

[51] Howell A.J., Buro K. (2009). Implicit beliefs, achievement goals, and procrastination: A mediational analysis. Learn. Individ. Differ..

[52] Lam TY P., Lau K.C. (2014). Examining factors affecting science achievement of Hong Kong in PISA 2006 using hierarchical linear modeling. Int. J. Sci. Educ..

[53] Ainley M., Ainley J. (2011). Student engagement with science in early adolescence: The contribution of enjoyment to students’ continuing interest in learning about science. Contemp. Educ. Psychol..

[54] (2022). Global Status Report on Physical Activity 2022.

[55] Cairney J., Kwan M.Y., Velduizen S., Hay J., Bray S.R., Faught B.E. (2012). Gender, perceived competence and the enjoyment of physical education in children: A longitudinal examination. Int. J. Behav. Nutr. Phys. Act..

[56] Tanaka C., Tanaka M., Tanaka S. (2018). Objectively evaluated physical activity and sedentary time in primary school children by gender, grade and types of physical education lessons. BMC Public Health.

[57] Carroll B., Loumidis J. (2001). Childrenís perceived competence and enjoyment in physical education and physical activity outside school. Eur. Phys. Educ. Rev..

[58] Gråstén A., Jaakkola T., Liukkonen J., Watt A., Yli-Piipari S. (2012). Prediction of enjoyment in school physical education. J. Sport. Sci. Med..

[59] Gao Z. (2008). Perceived competence and enjoyment in predicting students’ physical activity and cardiorespiratory fitness. Percept. Mot. Ski..

[60] Csikszentmihalyi M. (1975). Beyond Boredom and Anxiety.

[61] Logan S., Johnston R. (2009). Gender differences in reading ability and attitudes: Examining where these differences lie. J. Res. Read..

[62] Boerma I.E., Mol S.E., Jolles J. (2016). Teacher perceptions affect boys’ and girls’ reading motivation differently. Read. Psychol..

[63] Ericsson K.A., Krampe R.T., Tesch-Römer C. (1993). The role of deliberate practice in the acquisition of expert performance. Psychol. Rev..

[64] Marinak B.A., Gambrell L.B. (2010). Reading motivation: Exploring the elementary gender gap. Lit. Res. Instr..

[65] Britner S.L. (2008). Motivation in high school science students: A comparison of gender differences in life, physical, and earth science classes. J. Res. Sci. Teach. Off. J. Natl. Assoc. Res. Sci. Teach..

[66] Eccles J. (2011). Gendered educational and occupational choices: Applying the Eccles et al. model of achievement-related choices. Int. J. Behav. Dev..

[67] Ganley C.M., Vasilyeva M., Dulaney A. (2014). Spatial ability mediates the gender difference in middle school students’ science performance. Child Dev..

[68] Ahlqvist S., London B., Rosenthal L. (2013). Unstable identity compatibility: How gender rejection sensitivity undermines the success of women in science, technology, engineering, and mathematics fields. Psychol. Sci..

[69] Cheryan S., Ziegler S.A., Montoya A.K., Jiang L. (2017). Why are some STEM fields more gender balanced than others?. Psychol. Bull..

[70] Erdvik I.B., Øverby N.C., Haugen T. (2014). Students’ self-determined motivation in physical education and intention to be physically active after graduation: The role of perceived competence and identity. J. Phys. Educ. Sport.

[71] Kalaja S., Jaakkola T., Liukkonen J. (2010). Role of gender, enjoyment, perceived competence, and fundamental movement skills as correlates of the physical activity engagement of Finnish physical education students. Scandinavian Sport Studies Forum (SSSF).

[72] Shen B., Centeio E., Garn A., Martin J., Kulik N., Somers C., McCaughtry N. (2018). Parental social support, perceived competence and enjoyment in school physical activity. J. Sport Health Sci..

[73] Kovas Y., Garon-Carrier G., Boivin M., Petrill S.A., Plomin R., Malykh S.B., Spinath F., Murayama K., Ando J., Bogdanova O.Y. (2015). Why children differ in motivation to learn: Insights from over 13,000 twins from 6 countries. Personal. Individ. Differ..

[74] Dweck C.S. (2017). Mindset. Changing the Way You Think to Fulfil Your Potential.

